# Primary Cervicomedullary Junction Melanocytic Melanoma: An Illustrated Case Report

**DOI:** 10.1200/JGO.2016.005694

**Published:** 2016-07-06

**Authors:** Abhishek Mahajan, Rakesh Jalali

**Affiliations:** All authors, Tata Memorial Centre, Mumbai, India.

## CASE REPORT

**Fig 1:**
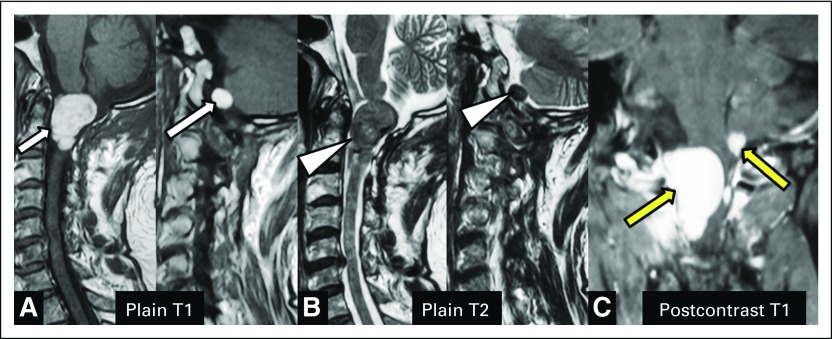
Images obtained before the second surgery (2012). Magnetic resonance imaging showed well-defined intradural extramedullary lesions at the cervicomedullary junction. On the plain T1-weighted sagittal view (A), the mass was hyperintense (white arrows); on the plain T2-weighted sagittal view (B), it showed hypo-intense signal intensity (arrowheads). The postcontrast T1-weighted coronal view (C) showed intense postcontrast enhancement (yellow arrows). The imaging findings described were diagnostic for melanoma.

**Fig 2:**
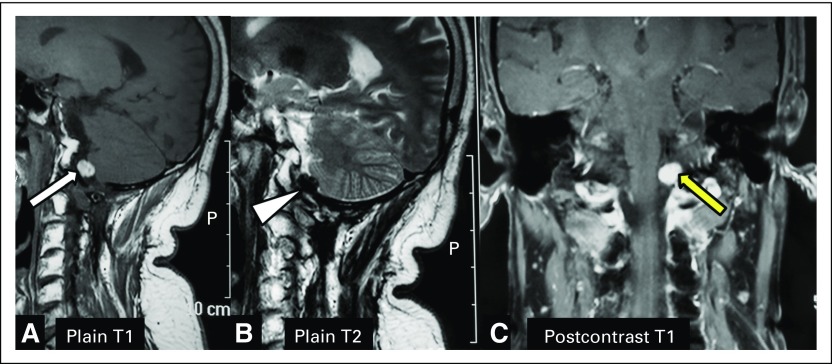
Follow-up images obtained after the second surgery (2014). Magnetic resonance imaging showed well-defined intradural extramedullary lesion at the cervicomedullary junction. On the plain T1-weighted sagittal view (A), the mass was hyperintense (white arrow); on the plain T2-weighted sagittal view (B), it showed hypo-intense signal intensity (arrowhead). The postcontrast T1-weighted coronal view (C) showed intense postcontrast enhancement (yellow arrow). The imaging findings described were diagnostic for melanoma and represented residual disease after the second surgery.

A 40-year-old man presented with a 6-month history of insidious and gradual onset of progressive pain in right shoulder and neck. He also noted progressive weakness of his right upper and lower limb. A magnetic resonance imaging (MRI) of his brain and spine was performed that revealed an intradural extramedullary lesion at the cervicomedullary junction (CMJ), for which he underwent surgery in February 2009. The histopathological diagnosis was melanocytic neoplasm of intermediate-grade malignancy. The symptoms reoccurred in October 2012, and a repeat MRI of the cervical spine was performed that revealed recurrent lesions at the CMJ. The patient was referred to our tertiary center for further management. Figure 1 shows the images obtained before the second surgery (2012), and Figure 2 displays the follow-up images obtained after the second surgery (2014). MRI showed well-defined intradural extramedullary lesions at the CMJ. On plain T1-weighted sagittal views (Figs 1A and 2A), the mass was hyperintense (white arrows); on plain T2-weighted sagittal views (Figs 1B and 2B), it showed hypointense signal intensity (arrowheads). Postcontrast T1-weighted coronal views (Figs 1C and 2C) showed intense postcontrast enhancement (yellow arrows). The imaging findings described were diagnostic for melanoma.

## DISCUSSION

Primary CNS melanoma is a rare entity, accounting for 1% of all melanoma cases and 0.06% to 0.1% of all CNS malignancies.^1,2^ Primary spinal cord malignant melanoma is even rarer, with most of the cases reported in the mid or lower thoracic spinal cord. Other pigmented CNS malignancies include meningeal melanocytoma, melanotic schwannoma, and blue nevus tumor of the CNS.^3^ Knowledge of the hallmark neuroimaging pattern is critical for appropriate and timely management.

MRI is the imaging modality of choice for characterizing tumors of the spinal cord; however, differentiation of the tumors on the basis of the morphology and signal-intensity pattern on MRI sometimes poses a diagnostic dilemma.^4,5^ The paramagnetic properties of melanin and intralesional hemorrhages in melanotic lesions lead to bright signal on T1-weighted and dark signal on T2-weighted imaging, which is described as the classic melanotic pattern. However, this characteristic appearance on imaging is not homogeneous and universal; rather, it is seen to depend on the percentage of melanocytes and prior hemorrhages. Lesions with > 10% melanin-containing cells tend to show this diagnostic imaging pattern, and the ones with < 10% tend to exhibit an amelanotic pattern (compared with cortex, hypo- or iso-intense on T1-weighted images, and hyper- or iso-intense on T2-weighted images).

Two more patterns have been described in literature; these are indeterminate or mixed pattern and hematoma pattern (in these patterns, the MR signal characteristics do not conform to either melanotic or amelanotic pattern).^6^ Also, it is important to note that on the basis of the MRI patterns, melanin cannot be completely distinguished from methemoglobin, and both have overlapping imaging morphology. This could be one of the possible explanations for hemorrhagic tumors masquerading as melanomas.^7^

Primary CNS melanomas are aggressive tumors and have grave prognoses. Surgery is the mainstay of treatment; complete resection has a better prognosis. Incompletely resected tumors have been found to benefit from temozolomide therapy. Immunotherapy, such as high-dose interferon beta or interferon alfa-2b, has been shown to improve disease control and survival; however, the toxicities related to the dosage of these drugs remain disputed.^8-10^ Our patient underwent complete excision of the tumor in 2009 and had recurrent disease in 2012, for which he had surgery twice. A centimeter-sized tumor (residual disease) on the left side was treated with external-beam radiotherapy (50.4 Gy in 28 fractions), followed by 12 cycles of temozolomide. The last follow-up was in April 2016, and interval MRI showed stable residual tumor.

To conclude, the MRI imaging characteristics described in our case highlight the typical radiologic findings in cases of primary CNS melanocytic tumors. Awareness of this imaging pattern aids in timely and appropriate diagnosis. Caution should be exercised in scenarios such as hemorrhagic cord tumors and melanotic schwannomas that may masquerade the typical MRI pattern of melanocytic tumor and may pose a diagnostic challenge to the radiologist.
